# The role of Neanderthal introgression in liver cancer

**DOI:** 10.1186/s12920-022-01405-7

**Published:** 2022-12-12

**Authors:** Angela M. Taravella Oill, Kenneth H. Buetow, Melissa A. Wilson

**Affiliations:** 1 Center for Evolution and Medicine, Arizona State University, Tempe, AZ USA; 2 School of Life Sciences, Arizona State University, Tempe, AZ USA

**Keywords:** Liver cancer, Introgression, Neanderthal, Metabolism, Immune function

## Abstract

**Background:**

Neanderthal introgressed DNA has been linked to different normal and disease traits including immunity and metabolism—two important functions that are altered in liver cancer. However, there is limited understanding of the relationship between Neanderthal introgression and liver cancer risk. The aim of this study was to investigate the relationship between Neanderthal introgression and liver cancer risk.

**Methods:**

Using germline and somatic DNA and tumor RNA from liver cancer patients from The Cancer Genome Atlas, along with ancestry-match germline DNA from unaffected individuals from the 1000 Genomes Resource, and allele specific expression data from normal liver tissue from The Genotype-Tissue Expression project we investigated whether Neanderthal introgression impacts cancer etiology. Using a previously generated set of Neanderthal alleles, we identified Neanderthal introgressed haplotypes. We then tested whether somatic mutations are enriched or depleted on Neanderthal introgressed haplotypes compared to modern haplotypes. We also computationally assessed whether somatic mutations have a functional effect or show evidence of regulating expression of Neanderthal haplotypes. Finally, we compared patterns of Neanderthal introgression in liver cancer patients and the general population.

**Results:**

We find Neanderthal introgressed haplotypes exhibit an excess of somatic mutations compared to modern haplotypes. Variant Effect Predictor analysis revealed that most of the somatic mutations on these Neanderthal introgressed haplotypes are not functional. We did observe expression differences of Neanderthal alleles between tumor and normal for four genes that also showed a pattern of enrichment of somatic mutations on Neanderthal haplotypes. However, gene expression was similar between liver cancer patients with modern ancestry and liver cancer patients with Neanderthal ancestry at these genes. Provocatively, when analyzing all genes, we find evidence of Neanderthal introgression regulating expression in tumor from liver cancer patients in two genes, *ARK1C4* and *OAS1*. Finally, we find that most genes do not show a difference in the proportion of Neanderthal introgression between liver cancer patients and the general population.

**Conclusion:**

Our results suggest that Neanderthal introgression provides opportunity for somatic mutations to accumulate, and that some Neanderthal introgression may impact liver cancer risk.

**Supplementary Information:**

The online version contains supplementary material available at 10.1186/s12920-022-01405-7.

## Background

Humans of non-African descent have about 2% Neanderthal DNA [1], with East Asians having about 20% more Neanderthal ancestry than Europeans [2]. Overall Neanderthal ancestry is depleted in functionally important regions of the genome, suggesting widespread purifying selection against Neanderthal DNA [3–5]. Nevertheless, archaic introgressed haplotypes have been retained in regions of human genomes associated with immune function [6–9] and metabolism [10]. Additionally, Neanderthal variants across humans are associated with disease and non-disease traits in humans [11–14], including cancer [14, 15]. Interestingly, by analyzing archaic introgression maps to detect polygenic patterns of adaptive introgression, a recent study found that genes relating to apoptosis and cell cycle—biological processes important in cancer risk—were enriched in archaic variants [16].

As the second leading cause of cancer death worldwide, liver cancer results in approximately 782,000 deaths globally per year (Bray et al. 2018). Hepatocellular carcinoma (HCC) is the most common type of liver cancer in adults. HCC diagnoses are increasing at an alarming rate across the world, likely due to the increase in incidences of common complex metabolic diseases, obesity, and non-alcoholic fatty liver disease (reviewed in [17]). In addition to metabolically derived causes, other risk factors include hepatitis virus infections (HBV and HCV) and alcohol consumption. A combination of differences in both environmental and genetic risk factors contributes to HCC risk variation across populations [18].

The liver plays an essential role in metabolism and in immunoregulation. The liver is the primary organ for metabolizing carbohydrates, fats, and proteins, and has important immunological functions, for example clearing pathogens that enter the blood while also maintaining immunotolerance [19]. In liver cancer, normal immune and metabolic functions are altered. For example, glycolysis and glycogen metabolism, nucleotide metabolism, amino-acid metabolism, and lipid metabolism are dysregulated in liver cancer (reviewed in [20]). Numerous metabolic genes are altered in HCC [21] and immune genes are down-regulated in HCC tumors compared to matched adjacent nontumorous tissue [22].

Interestingly, Neanderthal introgressed haplotypes are found in genes related to immune function and metabolism. For example, adaptive introgression has been observed in immune genes like *STAT2* in Melanesians [7], HLA Eurasians and Oceanians [6], *TLR6*-*TLR1*-*TLR10* cluster in Europeans and Asians [9, 23], and the OAS gene cluster in non-Africans [8, 24]. Neanderthal alleles affect gene expression levels in immune genes such as *OAS1/2/3* and *TLR1/6/10* [15, 25]. Further, in Europeans, regulatory variants introduced through Neanderthal introgression affect viral response [26]. Introgressed regions span genes associated with lipid metabolism and adipose tissue differentiation and distribution [10, 11, 27]. A GWAS on genetic risk factors associated with type-2 diabetes across individuals from Mexico and Latin America identified a novel introgressed locus spanning *SLC16A11* and *SLC16A13* associated with type 2 diabetes [11]. This study further found that *SLC16A11* may potentially play a role in hepatic lipid metabolism. The extent to which archaic introgressed regions contribute to other complex diseases, like liver cancer, is currently an open area of investigation.

To form a better understanding of the relationship between Neanderthal introgression and liver cancer risk, we have undertaken a systematic study of Neanderthal introgression in liver cancer using both germline and somatic DNA and tumor RNA from 177 individuals of European ancestry and 159 individuals of East Asian ancestry with liver cancer from The Cancer Genome Atlas (TCGA) [28], along with ancestry-match germline DNA from 503 individuals of European ancestry and 504 individuals of East Asian ancestry without a known cancer phenotype from the 1000 Genomes Resource [29], and allele specific expression (ASE) and gene expression data from normal liver tissue from 181 individuals of European ancestry from The Genotype-Tissue Expression (GTEx) project [30]. Among liver cancer patients, we find an overall enrichment of somatic mutations on Neanderthal introgressed haplotypes. We find higher expression of the Neanderthal alleles across genes where somatic mutations are enriched on Neanderthal introgressed haplotypes compared to genes without an enrichment of somatic mutations on introgressed haplotypes across both Europeans and East Asians but no evidence for differences in predicted functional consequences of somatic mutation on Neanderthal introgressed compared to modern haplotypes. In Europeans where we have ancestry-matched unaffected samples, generally we find that most genes show no difference in the relative expression of Neanderthal alleles between liver tumor and healthy liver tissue. There were two genes, *AKR1C1* and *OAS1*, though with significant differences in the relative expression of Neanderthal alleles between tumors from individuals with liver cancer and liver from unaffected individuals that also had overall gene expression differences between liver cancer patients with Neanderthal introgression and liver cancer patients without introgression. Lastly, in the analysis of germline DNA between liver cancer patients and the general population we find little differences in the proportion of Neanderthal introgression between liver cancer patients and the general population. Taken together, our results suggest that introgression provides opportunity for somatic mutations to accumulate, and that Neanderthal introgression may impact liver cancer risk in some genes.

## Methods

### Data

We used inferred archaic alleles found on introgressed segments in human genomes from [31] to identify introgression in individuals with liver cancer. We merged all putative archaic alleles from across all 1000 Genomes populations that were analyzed in [31] and retained alleles that matched the Altai Neanderthal genome and were absent or nearly absent in Yoruba (YRI) individuals from 1000 Genomes (Additional File 1: Fig. S1). Of the 798,688 putative archaic alleles, 251,733 matched the Altai Neanderthal genome. To match the genomic data used in this manuscript, we lifted over these Neanderthal introgressed SNPs from hg19 to GRCh38 using UCSC LiftOver tool [32]; of the 251,733 sites, 251,707 successfully lifted over and were used in the subsequent analyses.

To investigate patterns of introgression in individuals with liver cancer, we used aligned germline exome sequence data and bulk RNA sequence data from a total of 411 individuals that were processed in [33] from TCGA [28]. These data were aligned to a custom sex-specific version of GRCh38 using HISAT2 v.2.1.0 [34] based on the reported sex of the sample. Additionally for the RNA sequence data, *featureCounts* [35] was used to quantify gene level counts. For the exome data, A total of 66,983,203 SNPs across the autosomes were called using GATK’s v4.1.0.0 HaplotypeCaller and GenotypeGVCFs tools [36]. Biallelic SNPs were extracted using GATK’s v4.1.0.0 SelectVariants tool [36] and sites with a minimum mapping quality score of 30 and a 90% call rate across all samples were retained for our analyses. Site filtering was performed using bcftools v1.10.2 [37]. A total of 1,369,966 SNPs remained after filtering. We additionally used somatic variation data called using the MuTect2 pipeline from TCGA [28].

As a comparison to liver cancer patients, we used germline DNA data for individuals without a known cancer phenotype. To perform ancestry-matched comparisons with TCGA, we used variant data from the 1000 Genomes Release 3 mapped to GRCh38 [29] for 504 East Asian and 503 European samples. We also used WASP-corrected allele specific expression (ASE) tables for liver tissue from The Genotype-Tissue Expression (GTEx) Project version 8 [30].

Continental population ancestry was previously identified in the TCGA liver cancer set using PopInf [38]; we restricted our analyses here to individuals of European and East Asian ancestry and individuals of East Asian and European ancestry were analyzed separately in all subsequent analyses. There was a total of 177 samples called European ancestry and 159 samples called East Asian ancestry in the TCGA liver cancer data set. For GTEx, we assigned continental population ancestry using PopInf [38] here using whole exome VCFs from GTEx version 8 [30]. Across GTEx, only three samples had evidence of East Asian ancestry, so we restricted our analysis of ASE to individuals of European ancestry (Additional File 1: Fig. S2). A total of 181 individuals of European ancestry were retained for subsequent analysis.

All analyses presented here were performed at the gene level because the TCGA germline variants were from exome data. We downloaded all gene and gene predictions from NCBI RefSeq (GRCh37/hg19) using the UCSC table browser [39]. Gene coordinates were determined by taking the union of all the transcription starts and ends from all the isoforms of a gene.

### Analysis of somatic variation in liver cancer patients

To investigate whether, in individuals with liver cancer, somatic mutations are enriched in the presence of Neanderthal introgression, we tested whether somatic mutations are enriched or depleted on Neanderthal introgressed haplotypes compared to modern human haplotypes in genes with Neanderthal introgression. To identify whether somatic mutations were on Neanderthal introgressed haplotypes we merged each patients somatic mutations VCFs with the germline DNA using bcftools v1.10.2 merge utility [37] and plink v1.9 [40], and then phased this merged data using Beagle v5.2 [41] setting impute to false. After merging and phasing, 2,964,705 sites remained. With the phased data, we counted the number of somatic mutations on Neanderthal introgressed and modern haplotypes across genes using a custom python script. We first used bedtools intersect v2.27.1 [42] between the merged and phased VCF and tag SNP bed file to identify which sites in the VCF were introgressed. A haplotype was considered Neanderthal introgressed if there was at least one Neanderthal site with a matching Neanderthal allele. If there were no matching Neanderthal alleles across any sites on the haplotype, it was categorized as modern. For each gene where there were at least 5 haplotypes with Neanderthal introgression, a Fisher’s exact test was performed, in R [43], to test whether the proportion of haplotypes with somatic mutations is different between the Neanderthal introgressed and modern haplotypes. Correction for multiple testing was performed using the Bonferroni correction method. We also calculated a somatic mutation ratio for each individual across all introgressed genes [44]. Somatic mutation ratio was calculated as the total number of somatic mutations on Neanderthal introgressed haplotypes over the total length of the Neanderthal haplotypes over the total number of somatic mutations on the modern haplotypes over the total length of modern haplotypes.

To computationally assess the potential functional impact of somatic mutations on Neanderthal introgressed haplotypes, we ran Ensembl’s Variant Effect Predictor (VEP) tool [45] on the merged somatic/germline VCF. We used the "per_gene" flag to output the most severe consequence per gene. Using bedtools intersect [42], we then intersected the VEP results with the results table from the previous analysis that had which somatic mutations were on each haplotype for each gene. To assess whether the somatic mutations were more disruptive on Neanderthal introgressed haplotypes compared to modern haplotypes we summed the total number of predicted disruptive variants (high consequence VEP classification) and the total of variants that were predicted to not be disruptive (moderate, low, and modifier consequence VEP classification) for each gene across Neanderthal and modern haplotypes, separately. We then performed a Fisher’s Exact test, in R [43], to test whether the proportion of disruptive variants differed on Neanderthal introgressed and modern haplotypes. Correction for multiple testing was performed using the Bonferroni correction method.

### Allele specific and gene expression

To investigate whether Neanderthal introgressed alleles mediate gene expression in tumor tissue, we performed ASE analysis. The software WASP contains a set of tools that can correct for biases in allele-specific read data [46]. Prior to allele count quantification, we ran WASP’s remapping method on the tumor RNA sequence bam files to account for potential reference mapping bias. Of the 336 individuals of European and East Asian ancestry with germline DNA data, 330 individuals had bulk RNA sequence data derived from tumor. For each of these 330 liver cancer patients, we ran GATKs v4.1.0.0 ASEReadCounter tool [36] using the WASP corrected tumor RNA sequence bams and germline variants. We set a minimum depth of 10, minimum base quality of 10, and minimum read mapping quality of 10. After restricting to sites that overlapped with Neanderthal SNPs, there were a total of 4769 and 4925 heterozygous sites with a Neanderthal allele overlapping 550 and 542 genes in Europeans and East Asians, respectively.

For the Europeans only, where we had ancestry-match WASP corrected ASE tables from normal liver from GTEx, we compared the proportion of reads supporting the Neanderthal allele across genes in tumor and normal liver tissue. We removed genes with less than 5 heterozygous sites across samples and only analyzed genes that overlapped both TCGA and GTEx, resulting in 223 genes. To test whether there is a difference in the proportion of reads supporting the Neanderthal allele between tumors from liver cancer patients and normal liver from unaffected individuals, for each gene, we performed a Wilcoxon Rank Sum test in R [43]. Correction for multiple testing was performed using the Bonferroni correction method.

Gene-level count data was used to plot gene expression of tumor from in individuals with liver cancer (TCGA) and liver from unaffected individuals (GTEx) for genes identified as either having an enrichment of somatic mutations on Neanderthal introgressed haplotypes and/or significant difference in expression of the Neanderthal allele in tumor compared to unaffected liver. For each gene, CPM (counts per million) expression values were obtained using edgeR [47]. Samples were considered to have Neanderthal ancestry at a gene if they had at least one Neanderthal allele, while samples with no Neanderthal alleles at the gene were considered to have modern ancestry.

### Identifying Neanderthal introgression across genes in liver cancer patients and the general population

To investigate whether there is a relationship between Neanderthal introgression and liver cancer susceptibility, we compared the proportion of samples with evidence of Neanderthal introgression in individuals with liver cancer and non-affected individuals. For liver cancer patients, we used the exome germline variant data from TCGA, and for the non-affected group, we used variants from 1000 genomes. The Neanderthal introgressed SNPs described above were used to identify evidence of Neanderthal introgression at genes in liver cancer patients and non-affected samples. We kept Neanderthal SNPs that were present in the TCGA germline variant data using bedtools intersect v2.27.1 [42]. Of the 1,369,966 SNPs that were present in the filtered TCGA germline variant data, 9740 sites across 4179 genes overlapped Neanderthal SNPs.

For each gene, we identified whether samples had evidence of Neanderthal introgression using a custom python script. If a sample had a matching Neanderthal allele for at least one Neanderthal SNP that overlapped the gene, then that sample was called as having evidence of Neanderthal introgression at that gene. If a sample had no matching Neanderthal allele at all Neanderthal SNPs that overlapped the gene, then the sample was called as non-Neanderthal introgressed (or modern) at that gene. Counts were determined for both the TCGA sets and the 1000 genomes sets in this way. For each gene, a χ^2^ test was performed, in R [43], to test whether the proportion of samples with introgression was different between people with liver cancer and people not diagnosed with liver cancer. Correction for multiple testing was performed using the Bonferroni correction method.

All plotting and statistical analyses for the analyses presented here were performed in R [43]. All code generated and used for the analyses presented here can be found on GitHub: https://github.com/SexChrLab/Introgression_Cancer.

## Results

### Somatic mutations are enriched on Neanderthal introgressed haplotypes

**Fig. 1:**
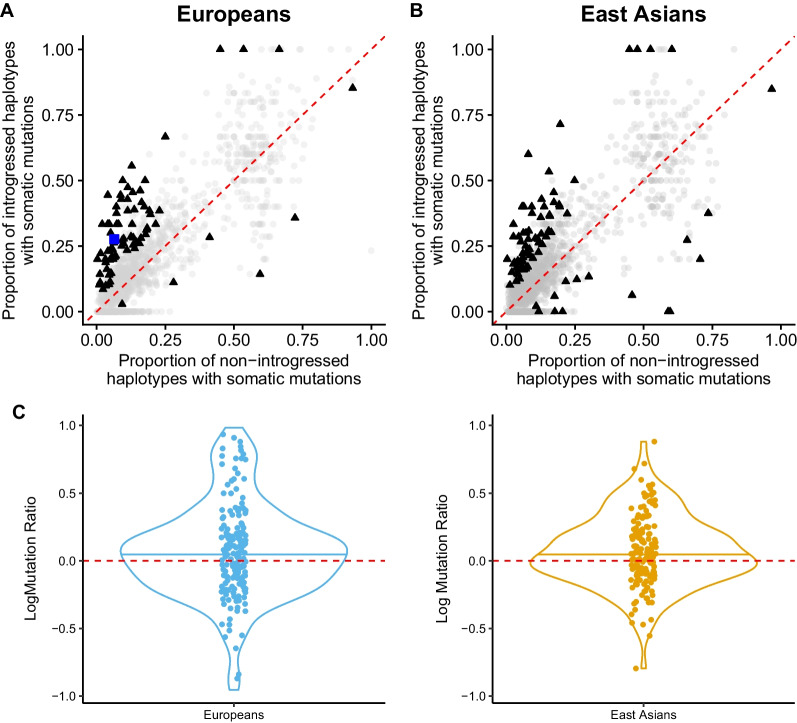
Across genes somatic mutations are enriched on Neanderthal introgressed haplotypes. Scatter plots of the genes that had an excess or depletion of somatic mutations on Neanderthal introgressed haplotypes in **A** Europeans and **B** East Asians and **C** violin plots of log somatic mutation ratio on Neanderthal introgressed haplotypes versus modern haplotypes in Europeans (blue) and East Asians (yellow). In **A** and **B** dots above the red dashed line represent genes that have a higher proportion of introgressed haplotypes with somatic mutations compared to non-introgressed haplotypes with somatic mutations. Dots below the red dashed line represent genes that have a lower proportion of introgressed haplotypes with somatic mutations compared to non-introgressed haplotypes with somatic mutations. Black triangles represent genes significant prior to multiple testing correction, blue squares represent genes significant after correcting for multiple testing, and grey circles represent genes that are not significant. In **C**, dots above the red dashed line represent liver cancer patients with a higher somatic mutation rate across Neanderthal introgressed haplotypes compared to modern haplotypes while dots below the red dashed line represent patients with a lower somatic mutation rate across Neanderthal introgressed haplotypes compared to modern haplotypes

Among liver cancer patients, we find an overall enrichment of somatic mutations in the presence of Neanderthal introgression. In genes with Neanderthal introgression in at least 5 haplotypes, we find an overall enrichment of somatic mutations on the Neanderthal introgressed haplotypes compared to the modern human haplotypes. Prior to multiple testing correction, there were 78 and 85 genes that had a significant difference in the proportion of somatic mutations between Neanderthal introgressed and modern haplotypes across Europeans and East Asians, respectively (Additional File 2: Tables S1 and S2). A majority of these genes—92% in Europeans and 81% in East Asians—showed a higher proportion of somatic mutations on the Neanderthal introgressed haplotypes compared to modern human haplotypes (Fig. 1). After correcting for multiple testing, one gene in Europeans, *PCSK9*, had significant enrichment of somatic mutations on the Neanderthal introgressed haplotypes compared to modern human haplotypes (*PCSK9*: *p*-value = 1.4 × 10^–5^, adjusted *p*-value = 0.019). For this gene, 28% of the Neanderthal introgressed haplotypes had somatic mutations, while 6% of the modern haplotypes had somatic mutations.

Similar to the per gene finding, we find that a majority of patients—58% of European and 59% of East Asian individuals—show a higher somatic mutation rate on the introgressed vs modern haplotypes (Fig. 1C; Additional File 2: Tables S3 and S4). We also performed a one-sided Wilcoxon rank sum test to test whether the median somatic mutation rate on the Neanderthal haplotypes was greater than on the modern haplotypes. We find that the somatic mutation rate on the Neanderthal haplotypes was significantly higher in East Asians (median mutation rate on Neanderthal haplotypes = 1.065 mutations per Mb, rate on modern haplotypes = 1.024 mutations per Mb, *p*-value = 0.049) and higher in Europeans (median mutation rate on Neanderthal haplotypes = 1.181 mutations per Mb, rate on modern haplotypes = 1.130 mutations per Mb) though *p*-value did not reach significance threshold (*p*-value = 0.056).

### Variant consequences of somatic mutations are similar between Neanderthal introgressed and modern haplotypes

Though we observe an overall pattern of enrichment of somatic mutations on Neanderthal haplotypes compared to modern haplotypes, we do not find evidence for an enrichment of disruptive somatic mutations—variants with a high consequence as determined by Variant Effect Predictor—on Neanderthal haplotypes compared to modern haplotypes. Of the 72 and 69 genes in Europeans and East Asians, respectively, with significant enrichment of somatic mutations on Neanderthal haplotypes compared to modern haplotypes (points above diagonal in Fig. 1B and C), no genes had a significant enrichment of high consequence somatic mutations on introgressed haplotypes compared to modern haplotypes (Additional File 2: Tables S5 and S6).

### There is higher expression of the Neanderthal alleles across genes where somatic mutations are enriched on Neanderthal introgressed haplotypes

**Fig. 2:**
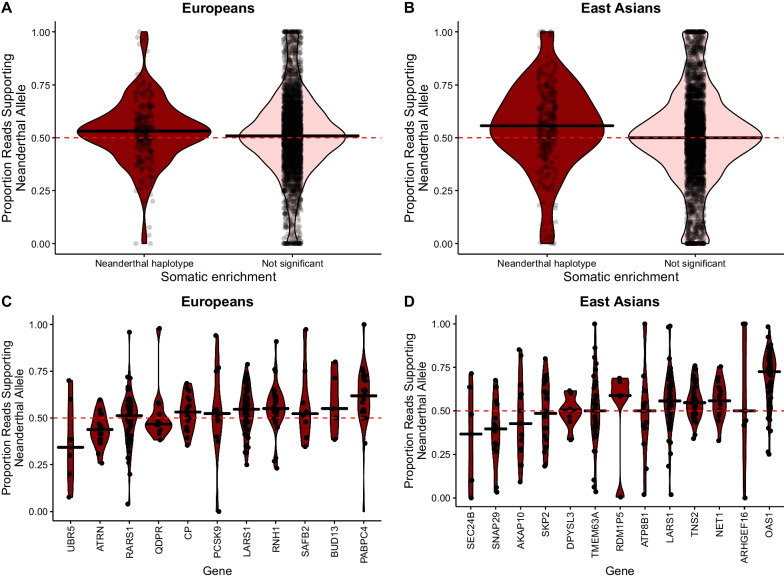
Genes with enrichment of somatic mutations on Neanderthal haplotypes have higher expression of Neanderthal alleles. Violin plots of the proportion of reads supporting the Neanderthal allele for all heterozygous introgressed sites for each gene, for each individual in **A** Europeans and **B** East Asians. Introgressed sites were grouped whether the site was in a gene where somatic mutations were enriched on Neanderthal haplotypes for that gene (dark red) or whether the site was in a gene that did not have an enrichment of somatic mutations on either the modern or Neanderthal haplotypes for that gene (light pink). We also plotted the proportion of reads supporting the Neanderthal allele for all heterozygous introgressed sites for each gene, for each individual grouped by the genes that had an enrichment of somatic mutations on introgressed haplotypes in **C** Europeans and **D** East Asians. Each point in these plots represents the proportion of reads supporting the Neanderthal allele for an individual at a particular gene; if an individual had more than one introgressed site within a gene, the proportion of reads supporting the Neanderthal allele was averaged across these sites. The median proportion of reads supporting the Neanderthal allele for each category and gene is shown as a solid horizontal line. Dashed red horizontal line represents equal expression of both the Neanderthal and modern allele

Allele specific expression refers to expression imbalance between two alleles at heterozygous sites. Imbalance of expression between two alleles at a site (deviation from equal expression of each allele) can influence gene expression and thus impact traits. If somatic mutations are regulating introgressed sequences, we expect to see differences in expression of Neanderthal alleles in the genes where we found an enrichment of somatic mutations on Neanderthal introgressed haplotypes. Overall, we find higher expression of Neanderthal alleles across genes that have an enrichment of somatic mutations on Neanderthal introgressed haplotypes compared to all other genes. We grouped Neanderthal introgressed sites based on whether they were in a gene that had an enrichment of somatic mutations on Neanderthal haplotypes, on modern haplotypes, or were not enriched on either Neanderthal or modern haplotypes. We find that the median proportion of reads supporting the Neanderthal allele is higher across genes that have an enrichment of somatic mutations on Neanderthal haplotypes compared to genes not enriched on either Neanderthal or modern haplotypes in both Europeans (median proportion of reads supporting the Neanderthal allele across genes with an enrichment of somatic mutations on introgressed haplotypes = 0.532, IQR = 0.150; median proportion for all other genes = 0.508, IQR = 0.202) and East Asians (median proportion of reads supporting the Neanderthal allele across genes with an enrichment of somatic mutations on introgressed haplotypes = 0. 557, IQR = 0. 241; median proportion for all other genes = 0.5, IQR = 0. 231; Fig. 2A and B). This observation was significant among East Asians (*p*-value = 8.05 × 10^–6^), but not among Europeans (*p*-value = 0.169). There were no Neanderthal introgressed sites with ASE in genes where we found an enrichment of somatic mutations on the modern haplotype.

Of the 236 and 223 genes with allele specific expression of Neanderthal alleles, 11 and 13 were genes that had enrichment of somatic mutations on Neanderthal introgressed haplotypes (Fig. 2C and D; Additional File 2: Tables S7 and S8). In Europeans, 8 genes showed an overall bias towards the Neanderthal allele (median proportion of reads supporting the Neanderthal allele greater than 0.5) while 3 genes showed an overall bias away from the Neanderthal allele (median proportion of reads supporting the Neanderthal allele less than 0.5) (Fig. 2C). In East Asians, 6 genes showed an overall bias towards the Neanderthal allele, 4 genes showed an overall bias away from the Neanderthal allele, and 3 genes showed no bias toward the modern or Neanderthal allele (Fig. 2D). Interestingly, introgressed sites in *PCSK9*—the one gene with significant enrichment of somatic mutations on the Neanderthal introgressed haplotypes compared to modern human haplotypes after multiple testing correction in Europeans—showed a slight overall bias towards the Neanderthal allele (median proportion of reads supporting the Neanderthal allele = 0.524 (IQR = 0.205); Fig. 2C).

### Most genes show no difference in the expression of Neanderthal alleles between tumors from individuals with liver cancer and liver from unaffected individuals

**Fig. 3:**
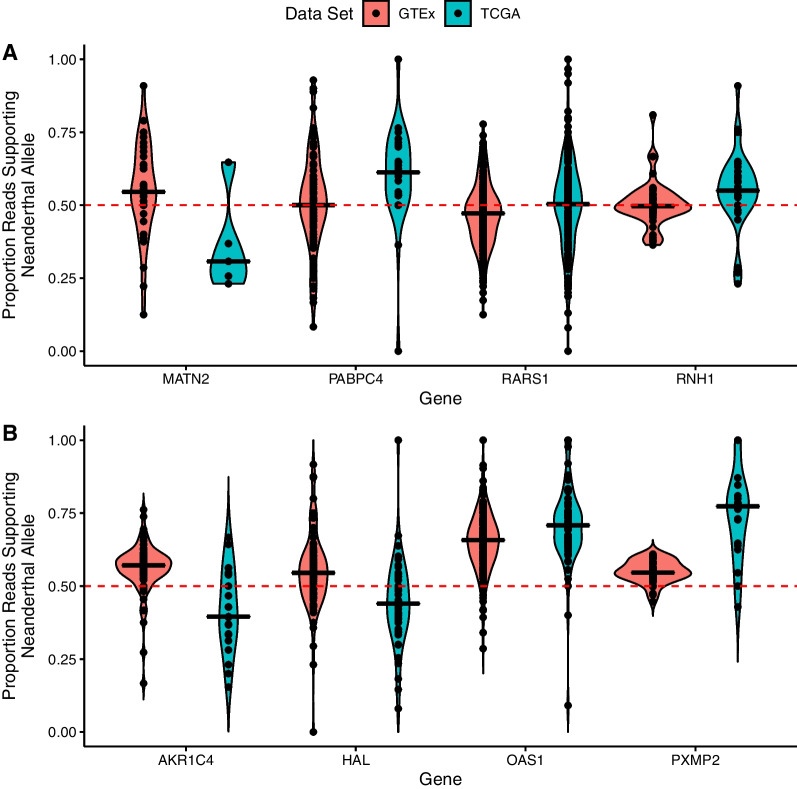
Genes with differences in expression of Neanderthal alleles between liver cancer patients and unaffected individuals. Violin plot of expression of Neanderthal alleles between tumor from individuals with liver cancer (TCGA, blue) and liver from unaffected individuals (GTEx, red). **A** Genes with a significant difference, prior to *p*-value adjustment, in the proportion of reads supporting the Neanderthal allele between TCGA and GTEx that also have an enrichment of somatic mutations on Neanderthal introgressed haplotypes, and **B** genes with significant differences, after *p*-value adjustment, in the proportion of reads supporting the Neanderthal allele between TCGA and GTEx

For Europeans, where we have ancestry-matched comparisons for liver tissue from unaffected individuals (GTEx), we find 33 out of 223 genes had a significant difference in the proportion of reads supporting the Neanderthal allele between tumors from liver cancer patients and liver from unaffected individuals, prior to multiple testing correction. Out of the 20 genes across Europeans that had both an excess of somatic mutations on Neanderthal introgressed haplotypes compared to modern haplotypes and allele specific expression data for Neanderthal alleles (Fig. 2C), four had a significant difference in the proportion of reads supporting the neanderthal allele between individuals from TCGA and GTEx, prior to multiple testing correction (*PABPC4:* *p*-value = 0.002, adjusted *p*-value = 0.388; *RARS1:* *p*-value = 0.001, adjusted *p*-value = 0.238; *RNH1:* *p*-value = 0.003, adjusted *p*-value = 0.612; *MATN2:* *p*-value = 0.037, adjusted *p*-value = 1; Fig. 3A; Additional File 2: Table S9). *PABPC4*, *RARS1* and *RNH1*, showed a pattern of higher expression of the neanderthal allele in TCGA compared to GTEx, while *MATN2*, showed a pattern of lower expression of the neanderthal allele in TCGA compared to GTEx (Fig. 3A). After *p*-value adjustment, four genes had a significant difference in the proportion of reads supporting the Neanderthal allele between tumors from liver cancer patients and liver from unaffected individuals (*AKR1C4*: *p*-value = 1.20 × 10^–5^, adjusted *p*-value = 0.003; *HAL*: *p*-value = 1.56 × 10^–6^, adjusted *p*-value = 0.0003; *OAS1*: *p*-value = 0.0001, adjusted *p*-value = 0.023; and *PXMP2*: *p*-value = 8.22 × 10^–6^, adjusted *p*-value = 0.002; Fig. 3B; Additional File 2: Table S9). *AKR1C4* and *HAL* showed a bias away from the Neanderthal allele in tumors, while *OAS1* and *PXMP2* showed a bias toward the Neanderthal allele in tumors (Fig. 3B).

**Fig. 4:**
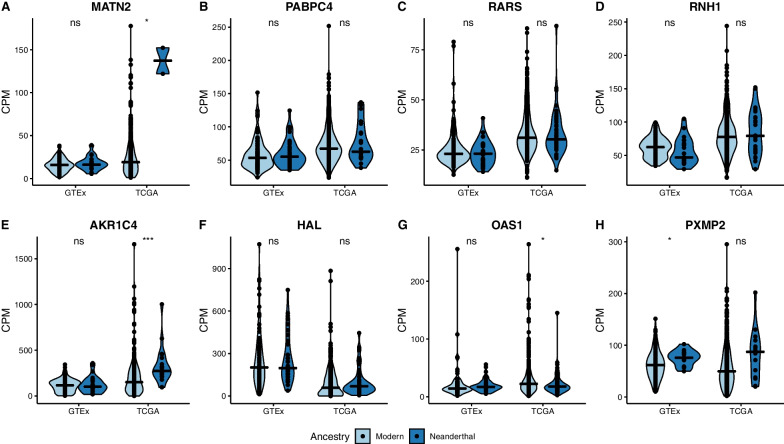
Gene expression is similar in samples with Neanderthal introgression and without Neanderthal introgression. Expression in counts per million (CPM) for genes with either **A**–**D** a significant enrichment of somatic mutation on introgressed haplotypes compared to modern haplotypes and a significant difference in expression of Neanderthal allele in tumor compared to unaffected liver prior to *p*-value adjustment, or **E**–**H** a significant difference in expression of Neanderthal allele in tumor compared to unaffected liver after *p*-value adjustment. For each gene, and each data set, we performed a Wilcoxon rank sum test between samples with introgression (Neanderthal, dark blue) and without (Modern; light blue). *p*-values were annotated for each comparison: ns *p* > 0.05, * *p* ≤ 0.05, ** *p* ≤ 0.01, *** *p* ≤ 0.001

For these significant genes, we investigated whether the change in expression of the Neanderthal allele resulted in overall gene expression differences. Within each dataset (tumor and normal) we look at expression in individuals with Neanderthal alleles (with Neanderthal introgression) and individuals with only modern alleles (without Neanderthal introgression) and test whether gene expression is different in the samples with Neanderthal introgression compared to without Neanderthal introgression (Fig. 4A–H). Across liver cancer patients, expression of *AKR1C4* was significantly higher in individuals with Neanderthal introgression (median CPM = 332.1) compared to individuals without (median CPM = 201.0; Fig. 4E). Additionally, expression of *OAS1* was lower in liver cancer patients with Neanderthal introgression (median CPM = 22.6) compared to liver cancer patients without introgression (median CPM = 32.3; Fig. 4G). Across unaffected liver, there was one gene, *PXMP2*, where expression was higher across individuals with introgression (median CPM = 75.7) compared to individuals without introgression (median CPM = 62.6; Fig. 4H).

### Most genes show no difference in Neanderthal introgression between liver cancer patients and the general population

**Fig. 5:**
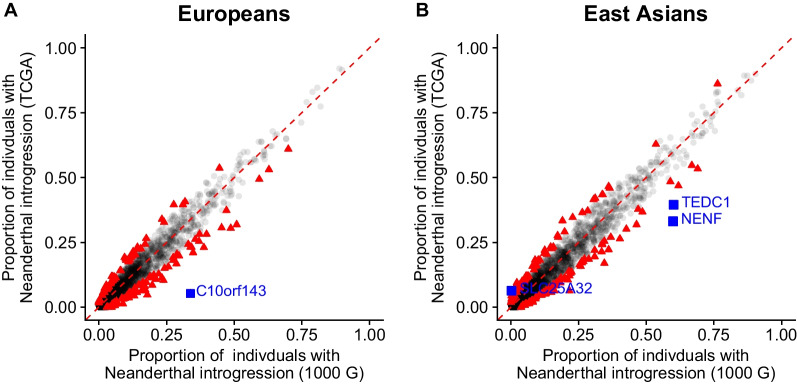
Liver cancer patients have little differences in proportions of Neanderthal introgression compared to non-affected individuals. Scatter plots of the proportion of liver cancer patients with Neanderthal introgression and the proportion of unaffected individuals with Neanderthal introgression for **A** European **B** and East Asian samples. Red triangle points represent significant genes before *p*-value adjustment (147 in European samples and 161 in East Asian samples) and blue square points are genes significant after multiple testing correction (1, in European samples and 3 in East Asian samples)

Overall, we find little differences in the proportion of Neanderthal introgression between liver cancer patients and the general population. Prior to multiple testing correction, 147 out of 3363 genes in individuals of European ancestry and 161 out of 3393 genes in individuals of East Asian ancestry had a significant difference in the proportion of Neanderthal introgression between liver cancer patients and unaffected individuals (Fig. 5; Additional File 2: Tables S10 and S11). These genes did not show a bias in directionality, as they were split approximately equally between enrichment and depletion of introgression (Fig. 5). After correcting for multiple testing, there was one gene with a significant depletion of Neanderthal introgression in European liver cancer patients compared to non-affected individuals—*C10orf143* (*p*-value = 7.12 × 10^–12^, adjusted *p*-value = 2.40 × 10^–8^; Fig. 5A). For Europeans, 5.2% of liver cancer patients have evidence of Neanderthal introgression at *C10orf143*, compared to 34% of non-affected individuals. In East Asians, there were two genes with a significant depletion of Neanderthal introgression in liver cancer patients compared to non-affected individuals—*NENF* (*p*-value = 3.32 × 10^–8^, adjusted *p*-value = 0.0001) and *TEDC1* (*p*-value = 1.11 × 10^–5^, adjusted *p*-value = 0.038; Fig. 5B). For East Asians, 33% of liver cancer patients have evidence of Neanderthal introgression at *NENF*, compared to 60% of non-affected individuals, and 39% of liver cancer patients have evidence of Neanderthal introgression at *TEDC1*, compared to 60% of non-affected individuals. There was additionally one gene with significant enrichment of Neanderthal introgression in East Asian liver cancer patients compared to non-affected individuals, *SLC25A32* (*p*-value = 1.03 × 10^–6^, adjusted *p*-value = 0.003; Fig. 5C), though was observed at a much lower frequency across both the liver cancer patients and the general population. At *SLC25A32*, 6.3% of liver cancer patients have evidence of Neanderthal introgression compared to 0.2% of non-affected individuals. Interestingly, the Neanderthal variants in these four of these genes are located in introns.

## Discussion

Here, we investigate the evidence for a relationship between Neanderthal introgression and liver cancer susceptibility. We leveraged publicly available Neanderthal introgressed SNPs along with previously generated germline and somatic DNA, and tumor RNA from patients with liver cancer from TCGA. Overall, our results suggest that Neanderthal introgression provides opportunities for somatic mutations to accumulate, and that some Neanderthal introgression may impact in liver cancer risk. We provide a summary table of genes discussed (Additional File 2: Table S12).

Among liver cancer patients, we find an overall enrichment of somatic mutations on Neanderthal introgressed haplotypes. This suggests that Neanderthal introgression may provide an opportunity for mutations to accumulate. A previous study found no difference in average mutation rate on Neanderthal haplotypes compared to on modern haplotypes since introgression [44]. Here, we analyzed somatic mutation rate in liver cancer patients across genes, finding examples where some genes have a higher proportion of somatic mutations on Neanderthal compared to modern haplotypes and observe an overall pattern of higher somatic mutation rate on Neanderthal compared to modern haplotypes. Additionally, across the genes we assessed, we find a median somatic mutation rate around 1 mutation per Mb, with values ranging from 0.35 to 12.20 mutations per Mb across individuals (Additional File 2: Tables S3 and S4). This is in line with what others have reported [48, 49]. For example, one study found an average mutation rate of 3.69 per Mb in HCC with a range of 0.07–39 across samples [48]. It is important to note, however, that not all somatic mutations contribute to cancer, so the observation of somatic mutations enriched on Neanderthal introgressed haplotypes alone does not imply an impact on liver cancer etiology.

One gene, *PCSK9*, has a significantly higher proportion of somatic mutations on Neanderthal introgressed haplotypes compared to modern haplotypes after multiple testing correction in Europeans. *PCSK9*, proprotein convertase subtilisin/kexin type 9, is involved in cholesterol and fatty acid metabolism [50], and has been linked to various cancers, including hepatocellular carcinoma (reviewed in [51]). In hepatocellular carcinoma, *PCSK9* has decreased expression compared to adjacent liver tissue [52]. *PCSK9* is also protective against HCV infection—an important risk factor for HCC [53]. Given previous reports of introgression’s importance in metabolism [10, 11] and in immunity [6, 7, 9, 23–25], it is possible that introgression in this region could be important in either metabolic- or immune-related processes and subsequently contribute to liver cancer susceptibility. This gene did not show a difference in allele-specific expression of the Neanderthal allele between patients with liver cancer and unaffected individuals with a median allele balance of 0.524 in liver cancer patients and 0.522 in unaffected individuals, suggesting that the somatic mutations are largely not affecting regulation of *PCSK9*. Instead, these mutations do not appear to have a functional effect as all of the somatic mutations in this gene had a modifier impact and were located in noncoding regions.

We found no difference in variant consequences of somatic mutations on Neanderthal haplotypes compared to on modern haplotypes, suggesting the somatic mutations in these introgressed regions are likely not resulting in protein level changes. These results are similar to what has been observed among Icelanders, where no enrichment of deleterious variants on archaic haplotypes compared to modern haplotypes were observed [44].

Among Europeans, there were four genes with both an enrichment of somatic mutations on Neanderthal introgressed haplotypes and significant differences in the proportion of reads supporting the of Neanderthal allele between patients with liver cancer and unaffected individuals prior to multiple testing correction—*PABPC4*, *MATN2, RARS1*, and *RNH1*. Of these, *PABPC4* and *MATN2* have been shown previously to be involved in liver cancer. *PABPC4*, poly(A) binding protein cytoplasmic 4, is involved in translation and mRNA stability [54]. Increased expression of *PABPC4* increases multiple cancer stem cell populations and cancer stem cell-related features in HCC cells [55]. Overexpression of lncRNA RP11-286H15.1 inhibits proliferation and metastasis of HCC cells by promoting ubiquitination of PABPC4 [56]. We found elevated expression of Neanderthal alleles in liver tumor samples compared to liver from unaffected individuals in *PABPC4*. It is possible that somatic mutations may be manipulating the Neanderthal introgressed haplotype resulting in more expression of this gene. However, we did not find a difference in expression of *PABPC4* between liver cancer patients with Neanderthal and liver cancer patients without Neanderthal introgression at this gene. Additionally, most of the somatic mutations on the Neanderthal haplotype were in non-coding regions, with the exception of 2 missense mutations, so it is likely that some somatic mutations are not altering expression of *PABPC4*.

*MATN2,* Matrilin-2, encodes an extracellular adaptor protein that plays an important role in promoting regeneration of different tissues like skeletal muscle and liver, skin wound healing, Schwann cell migration, neurite outgrowth, and neuromuscular junction formation [57]. We found that expression of the Neanderthal *MATN2* allele was significantly lower than the modern human allele in liver cancer compared to unaffected livers. *MATN2* has increased expression in tumors compared to normal liver [58]. It is possible that the Neanderthal haplotype may impact *MATN2* expression. Consistent with this, we observed higher expression of *MATN2* in liver cancer patients with Neanderthal introgression compared to liver cancer patients without Neanderthal introgression. However, there were only 2 samples with Neanderthal introgression and expression data, and the expression values fell within values observed in liver cancer patients without introgression at this gene. Further, none of the somatic mutations on the Neanderthal introgressed haplotypes in this gene were in coding regions. Taken together, these results suggest that introgression at this gene may not impact expression of *MATN2*.

The two other genes, *RARS1* and *RNH1*, have shown to be involved in other cancers, but not explicitly liver cancer. Here we found that *RARS1* had significantly higher expression of the Neanderthal allele in liver cancer compared to unaffected liver, with expression biased away from the Neanderthal allele compared to the modern allele in unaffected liver and roughly equal expression of the Neanderthal and modern alleles in liver cancer patients. *RARS1*, arginyl-tRNA synthetase 1, plays a role in protein synthesis [59] and higher expression of this gene was observed in pituitary adenomas compared to normal pituitary [60]. It is possible that the Neanderthal haplotype may impact expression of *RARS1* in liver cancer. However, expression of *RARS1* is similar in liver cancer patients with Neanderthal introgression compared to liver cancer patients without Neanderthal introgression in this gene. Most of the somatic mutations on the Neanderthal introgressed haplotypes in *RARS1* were in noncoding regions (there was one synonymous variant), so likely somatic mutations are not affecting regulation of *RARS1*. *RNH1*, ribonuclease/angiogenin inhibitor 1, is important in promoting processing of an miRNA (miR-21) associated with features important in cancer like migration, cell proliferation, invasion, anti-apoptosis, and metastasis [61, 62]. Additionally, *RNH1* is highly expressed in histone deacetylase inhibitor resistant gastric cancer cell lines [63]. For *RNH1*, we found that expression of the Neanderthal allele was significantly higher than the modern human allele in liver cancer compared to unaffected liver. Again, expression was similar in liver cancer patients with Neanderthal introgression and liver cancer patients without Neanderthal introgression, and most of the somatic mutations were in noncoding regions, with the exception of a missense and a synonymous variant, suggesting that the somatic mutations are largely not affecting regulation of *RNH1*. Taken together, though these genes are enriched for somatic mutations, and we observe allele expression differences of Neanderthal alleles between tumor and normal, Neanderthal introgression is likely not influencing this, as most of the somatic mutations were in non-coding regions and overall gene expression patterns were similar in individuals without Neanderthal introgression.

We also found four genes in Europeans that had a significant difference, after *p*-value adjustment, in the proportion of reads supporting the Neanderthal allele between tumors from liver cancer patients and liver from unaffected individuals—*OAS1*, *AKR1C4, PXMP2*, and *HAL*. They also had a higher proportion of somatic mutations on Neanderthal haplotypes but did not reach significance. All of these genes have been previously linked to different cancers. *OAS1* encodes a protein that promotes degradation of viral RNA and prohibits viral replication [64]. This gene is part of a cluster of genes (OAS gene cluster—*OAS1/2/3*) that has been previously shown to have evidence of adaptive introgression in Europeans by possibly playing a functional role in the innate immune response [25]. High expression of *OAS1* is correlated with worse prognosis for individuals with breast cancer [65] and a SNP within *OAS1* is associated with prostate cancer [66]. In our analysis we find higher expression of the Neanderthal allele in tumor from liver cancer patients compared to liver from unaffected individuals and lower overall expression of *OAS1* in liver cancer patients with introgression compared to liver cancer patients without introgression, suggesting introgression may be affecting expression at *OAS1* in liver cancer. With the exception of two missense variants, all of the somatic mutations on the Neanderthal haplotype were in noncoding regions, suggesting somatic mutations are not affecting regulation of *OAS1*. Taken together, though Neanderthal introgression in *OAS1* may provide a benefit to the human immune system [25], given its role in other cancers, it is possible that introgression in this gene may be contributing to liver cancer susceptibility. Our results highlight the complexity of evolutionary consequences from archaic introgression.

*AKR1C4*, aldo- keto reductase family 1 member C4, is involved in NADPH-dependent reduction and plays an important role in metabolism of steroid hormones [67]. Expression of this gene is localized in the liver [68]. Other genes in this family (*AKR1C1-3*) have been associated with different cancers including liver cancer [69–71]; however the role of *AKR1C4* in liver cancer is still unclear as expression level difference between tumor and normal tissue were found to be inconsistent across different databases [71]. Here, we find expression of the Neanderthal *AKR1C4* allele was significantly lower than the modern human allele in liver cancer compared to unaffected livers. It is possible that introgression is impacting *AKR1C4* expression in liver cancer. Consistent with this, we find higher expression of *AKR1C4* in liver cancer patients with Neanderthal introgression compared to liver cancer patients without Neanderthal introgression in this gene, suggesting introgression may be affecting expression at *AKR1C4* in liver cancer. All of the somatic mutations on the introgressed haplotypes were in non-coding regions though, with the exception of one missense variant, so it is likely that somatic mutations are not affecting regulation of *AKR1C4*. Overall, our results suggest that Neanderthal introgression regulates expression of AKR1C4 in tumor and this may contribute to liver cancer etiology.

*PXMP2*, peroxisomal membrane protein 2, is a channel-forming protein in mammalian peroxisomes [72]. In a co-expression network analysis in esophageal squamous cell cancer, *PXMP2* was found to be a hub gene possibly linked to lipid metabolism and potentially playing a role in esophageal squamous cell cancer progression [73]. We found that expression of the Neanderthal *PXMP2* allele was significantly higher than the modern human allele in liver cancer compared to unaffected livers. However, there was no difference in expression of *PXMP2* in liver cancer patients with Neanderthal introgression and liver cancer patients without Neanderthal introgression in this gene. Additionally, all of the somatic mutations on the Neanderthal haplotypes were in non-coding regions, except one missense variant, suggesting that somatic mutations are not affecting regulation of PXMP2.

*HAL*, histidine ammonia-lyase, encodes an enzyme that plays a role in histidine catabolism [74]. Higher levels of *HAL* expression were found among leukemia patients with higher survival rates [75]. We find significantly lower expression of the Neanderthal allele in liver cancer compared to unaffected livers and overall lower expression of *HAL* in liver cancer and unaffected livers. However, a similar pattern of overall gene expression was observed in liver cancer patients without Neanderthal introgression at *HAL*. Additionally, the somatic mutations on the introgressed haplotypes were in noncoding regions, except for one synonymous variant, suggesting somatic mutations are not affecting regulation of *HAL*.

For the candidate genes in our analysis—genes where Neanderthal introgression might be impacting gene expression in tumors (*AKR1C4* and *OAS1*) —we looked at the Altai Denisovan allele at the introgressed SNPs characterizing the Neanderthal haplotype. For *AKR1C4*, all four of the archaic alleles in this gene matched the Altai Neanderthal but not the Altai Denisovan allele. Similarly, for *OAS1*, all three of the archaic alleles in this gene matched the Altai Neanderthal but not the Altai Denisovan allele. Thus, these archaic alleles are likely Neanderthal-specific introgressed variants, rather than archaic in general.

In the analysis of germline DNA, we find little differences in the proportion of Neanderthal introgression between liver cancer patients and the general population, with the exception of three genes in East Asians and one gene in Europeans. This suggests that broadly there is not a genome-wide effect of Neanderthal introgression on liver cancer susceptibility but that some introgression may contribute to liver cancer susceptibility. In Europeans, *C10orf143* had a lower proportion of Neanderthal introgression among liver cancer patients compared to the general population and in East Asians, *NENF* and *TEDC1* showed a similar pattern. The enrichment of Neanderthal introgression in the general population suggests that Neanderthal introgression may be protective in these regions. However, the Neanderthal variants in these genes are located in introns, there was no allele specific expression data for the Neanderthal alleles in these genes, and the somatic mutations on the Neanderthal introgressed haplotypes were in non-coding regions, suggesting these genes might not have a functional effect.

In East Asians, *SLC25A32* had a higher proportion of Neanderthal introgression among liver cancer patients compared to the general population. The depletion of Neanderthal introgression in the general population suggests that introgression may be contributing to liver cancer risk in this region. However, similar to the other three significant genes in this analysis, the Neanderthal variant in this gene is located in an intron and there were no somatic mutations in coding regions, so also likely not having a functional effect.

## Conclusion

We investigate the evidence for a relationship between Neanderthal introgression and liver cancer risk. We report a pattern of enrichment of somatic mutations on Neanderthal introgressed haplotypes, suggesting that Neanderthal introgressed regions may be more permissive to somatic mutation. We also find evidence of Neanderthal introgression influencing gene regulation in tumor, suggesting some introgression may impact liver cancer susceptibility.

## Supplementary Information

**Additional file 1: Fig. S1.** Neanderthal alleles were conditioned on being low frequency in Africans. Frequency distribution of the Neanderthal alleles leveraged in our analyses across Yoruba individuals from 1000 Genomes. **Fig. S2.** Identifying continental ancestry in GTEx. PopInf results from 226 individuals from GTEx version 8. Circles are female samples, squares are male samples. Plus sign is the centroid coordinate for the reference population panel samples (in grey), circles are 1, 2, and 3 standard deviations from the centroid.

**Additional file 2: Table S1.** Fisher’s exact test results for the European set. **Table S2.** Fisher’s exact test results for the East Asian set. **Table S3.** Somatic mutation ratio results for the European set. **Table S4.** Somatic mutation ratio results for the East Asian set. **Table S5.** Summarized Variant Effect Predictor results on Neanderthal and modern haplotypes for the European set. **Table S6.** Summarized Variant Effect Predictor results on Neanderthal and modern haplotypes for the East Asian set. **Table S7.** Allele specific expression of Neanderthal alleles for genes in Europeans. Median proportion of reads supporting the Neanderthal alleles in each gene with at least 5 samples. Each gene is also annotated with whether the gene shows enrichment of somatic mutations on Neanderthal haplotypes or was not significant. **Table S8.** Allele specific expression of Neanderthal alleles for genes in East Asians. Median proportion of reads supporting the Neanderthal alleles in each gene with at least 5 samples. Each gene is also annotated with whether the gene shows enrichment of somatic mutations on Neanderthal haplotypes or was not significant. **Table S9.** Wilcoxon Rank Sum test results for the genes with Neanderthal allele expression in both tumor and unaffected liver from European samples. **Table S10.** χ^2^ test results for the European set. **Table S11.** χ^2^ test results for the East Asian set. **Table S12.** Summary of genes mentioned in discussion.

## Data Availability

All code generated and used for the analyses presented here can be found on GitHub: https://github.com/SexChrLab/Introgression_Cancer. TCGA liver cancer data can be found on dbGap at https://www.ncbi.nlm.nih.gov/gap/ (dbGaP Accession: phs000178) and NCI Genomic Data Commons at https://portal.gdc.cancer.gov/projects/TCGA-LIHC. GTEx data can be found on dbGap (dbGaP Accession: phs000424.v8.p2). The 1000 Genomes data can be found at http://ftp.1000genomes.ebi.ac.uk/vol1/ftp/data_collections/1000_genomes_project/release/20181203_biallelic_SNV/.

## Abbreviations

TCGA: The cancer genome atlas
ASE: Allele specific expression
GTEx: Genotype-tissue expression
VEP: Variant effect predictor
HCC: Hepatocellular carcinoma
HBV: Hepatitis B
HCV: Hepatitis C
CPM: Counts per million
