# Supplementary material for: The role of Neanderthal introgression in liver cancer
Source: BMC Med Genomics. 2022 Dec 12;15:255. doi: 10.1186/s12920-022-01405-7 (PMC9743633; doi:10.1186/s12920-022-01405-7)

**Additional File 1: Figure S1. Neanderthal alleles were conditioned on being low frequency in Africans.** Frequency distribution of the Neanderthal alleles leveraged in our analyses across Yoruba individuals from 1000 Genomes.

**Figure S2. Identifying continental ancestry in GTEx.** PopInf results from 226 individuals from GTEx version 8. Circles are female samples, squares are male samples. Plus sign is the centroid coordinate for the reference population panel samples (in grey), circles are 1, 2, and 3 standard deviations from the centroid.


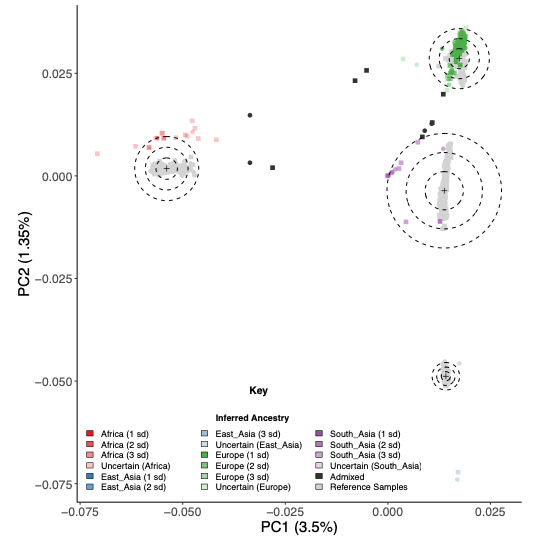

Supplement: Supplementary file 1 — Additional file 1: Fig. S1. Neanderthal alleles were conditioned on being low frequency in Africans. Frequency distribution of the Neanderthal alleles leveraged in our analyses across Yoruba individuals from 1000 Genomes. Fig. S2. Identifying continental ancestry in GTEx. PopInf results from 226 individuals from GTEx version 8. Circles are female samples, squares are male samples. Plus sign is the centroid coordinate for the reference population panel samples (in grey), circles are 1, 2, and 3 standard deviations from the centroid. [file 12920_2022_1405_MOESM1_ESM.docx]
